# General and sport-related marketing techniques in Canadian recreation and sport facilities: cross-sectional photo analysis of food and beverage advertisements

**DOI:** 10.1017/S1368980026102377

**Published:** 2026-03-26

**Authors:** Nan Lei, Melanie Warken, Amanda Michael, Tammy Benteau, Rachel Joyce Lian Prowse

**Affiliations:** 1 Memorial University of Newfoundland, Faculty of Medicine, St. John’s, Canada

**Keywords:** Food, Marketing, Child, Adolescent, Sports, Recreation, Canada

## Abstract

**Objective:**

To evaluate food marketing techniques used in Canadian recreation and sport facilities and assess the healthfulness of foods and beverages marketed by the techniques.

**Design:**

Cross-sectional content analysis of photographed food marketing instances coded for marketing techniques according to Health Canada’s Monitoring Protocol, developed for monitoring food marketing techniques across settings, supplemented with new inductively identified codes and sport-related marketing techniques. Healthfulness was classified as ‘of concern’ or ‘not of concern’ according to cut-offs of sodium, sugar and saturated fat established by Health Canada.

**Setting:**

Recreation and sport facilities in Canada

**Participants:**

134 facilities with 2576 food marketing instances

**Results:**

91·4 % of food marketing instances included at least one general marketing technique. Branded infrastructure, displays and furniture was the most prevalent (87·9 %) and appeared with another technique half of the time. Sport-related marketing appeared in 12·2 % of marketing instances, with most referring to sponsors. Most (86·5 %) marketing instances were ‘of concern’. Food marketing instances with sport-related marketing (97·6 %) were significantly more likely to be ‘of concern’ than without sport-related marketing (84·6 %) (χ^2^ = 20·54, *P* < 0·001). Three new indicators – appeals to taste, appeals to emotion, and cross-channel references – captured persuasive elements not addressed by the current monitoring protocol.

**Conclusions:**

This study highlights the presence of food branding and the use of sport-related marketing to promote unhealthy products/brands in recreation and sports facilities. Monitoring protocols may underestimate exposure to persuasive food marketing by overlooking subtle, symbolic and cross-channel techniques. Future research can be improved by including subtle techniques and reinforced messages across marketing channels.

Dietary behaviours in Canada are shaped by a combination of structural and social factors, including food availability, pricing, social norms and food marketing^(1)^. Among these, food marketing is a powerful influence that shapes food preferences and consumption patterns, particularly among children – a population highly susceptible to persuasive messaging. Monitoring food marketing across settings is recommended to understand food marketing exposures and their potential to widely influence diets^(2)^. In Canada, food marketing exposures are multiple and occur across various settings, including digital devices (television, online, social media), retail stores and restaurants, product packaging, schools, and recreation and sports facilities (RSF).

RSF are widely used by children and families for organised physical activity, yet the dominance of unhealthy food and beverage marketing within these settings stands in contrast to their role as health-supportive environments^(2,3)^. A previous study has reported higher consumption of fast food and sugar-sweetened beverages among adolescents engaged in organised sports, which may reflect extended time spent in RSF and the normalisation of ‘arena foods’ as part of the sport experience^(4)^. This pattern may be reinforced by structural features of RSF, including limited access to nutritious options and the pervasive presence of unhealthy food sponsorship^(5,6)^. These structural conditions are enacted and sustained through specific marketing strategies that promote unhealthy products within RSF.

How food and beverage products are promoted within RSF may further influence consumer perceptions – particularly among children and families – by linking unhealthy products with sport and physical activity^(7)^. Common promotional strategies include branded signage, team sponsorship, product vouchers and logo placements on equipment or apparel^(8)^. These strategies may reinforce a misleading ‘health halo’ effect, whereby unhealthy products are perceived as healthier due to their association with physical activity and sport^(9)^. Such misperceptions may be further shaped by contextual cues in RSF, as consumers – including parents – often infer product healthfulness based on setting rather than nutritional content^(10)^. Emerging evidence also suggests that sport-linked branding – such as athletic slogans or performance cues – can enhance perceived healthfulness while obscuring nutritional risks, particularly among young audiences^(11)^.

The WHO recommends that child-oriented settings – including sport venues – be free from the marketing of unhealthy food and beverages^(12)^. Yet, no research has investigated the presence of food marketing techniques used in recreation and sport facilities, which reveals a gap in knowledge to generate relevant and effective policies. In Canada, national efforts have been made to monitor food marketing to children^(13)^. The Food and Beverage Marketing Monitoring Framework recently established by Health Canada was developed through extensive literature review and expert consultation, with the goal of tracking both the frequency and power of food marketing across settings and media^(14)^. It reflects an important step toward systematically documenting children’s and families’ exposure to persuasive marketing strategies, including in community environments.

This study aims to use Health Canada’s Monitoring Food and Beverage Marketing to Children Protocol to evaluate the use of food marketing techniques in RSF in Canada. We aimed to examine the persuasive content of food marketing in a diverse sample of Canadian RSF using previously collected data. Specifically, we aimed:

- To quantify the use of marketing techniques
- To assess the use of sport-related marketing techniques and its use combined with general marketing techniques
- To evaluate the use of marketing techniques by product/brand healthfulness.

## Methods

### Study design

This cross-sectional study evaluated food and beverage marketing in Canadian RSF through content coding of photographic records collected during on-site audits and focused on the prevalence, characteristics and healthfulness of general and sport-related marketing techniques, as defined by Health Canada’s protocol for monitoring food marketing to children^(15)^.

### Setting and sample

Eligible RSF were identified from Statistics Canada’s Open Database of Recreation and Sport Facilities, supplemented by stakeholder lists and targeted web searches^(16)^. Inclusion criteria were as follows: (1) indoor, publicly accessible venues; (2) those offering structured sport or recreation programming for children under 18 years of age in popular sports such as ice hockey, swimming and soccer; and (3) located below the fifty-fifth parallel and off reserve land. Facilities were excluded if they consisted of outdoor leisure spaces, lacked organised child-focused programming, were closed seasonally during the February–May 2022 data collection window or were located within primary schools. Indoor facilities hosting organised programming in these sports were selected because they provide the most consistent and repeated exposure to food marketing for children, whereas outdoor leisure spaces do not reflect comparable exposure environments. Facilities located above the fifty-fifth parallel and on reserve land were not included due to lower population densities and the distinct recruitment processes required for facilities located on reserve land.

Facility managers/directors (*n* 341) were randomly invited to participate through phone and email invites, with up to three follow-ups. A total of 137 RSF agreed to participate, but after three withdrew from the study, the final sample included 134. Participating facilities received a $25 e-gift card as an incentive. This study was conducted according to the guidelines laid down in the Declaration of Helsinki, and all procedures involving research study participants were approved by the institutional research ethics boards at Memorial University of Newfoundland (20220510), Public Health Ontario, Dalhousie University (2021-5850) and the Universities of Alberta (Pro00105324), Calgary (REB20-0415) and Saskatchewan (BEH 16-314).

### Data collection

On-site audits were conducted by trained local research assistants using the validated Food and Beverage Marketing Assessment Tool for Settings^(17)^. Food and Beverage Marketing Assessment Tool for Settings is a validated audit tool developed to systematically assess food and beverage marketing in recreation and sport facilities, capturing both the frequency (exposure) and content features (power) of marketing through structured observation and photographic documentation^(17)^.

Food marketing was defined as any commercial promotion, advertising or messaging related to food, beverages, brands or retailers, excluding packaging. Observations of food marketing were recorded on the Food and Beverage Marketing Assessment Tool for Settings and photographed. Across the participating facilities, 3521 food marketing instances were recorded. Of these, 942 instances (26·8 %) were excluded from this study due to missing/unclear photographs, and three were removed for being non-commercial food marketing. The final analytic sample included 2576 marketing instances.

### Marketing technique content analysis

Each marketing instance was independently coded by two trained research assistants for the presence or absence of predefined indicators, allowing multiple indicators per instance. Discrepancies were resolved through discussion and consensus with a third rater, with advice from experts at Health Canada familiar with the coding protocol for refinement and consistent implementation of the protocol.

The coding framework included thirty-one general indicators^(15)^ with emphasis on explicit, child-directed techniques – such as characters, premiums or promotional language. The coding framework provided less guidance on capturing less overt strategies like emotional appeals or sport-linked branding, which are prominent in RSF^(10)^. As a result, certain promotional dynamics in RSF may be overlooked despite their potential impact on children’s real-world exposure. Additional unique indicators not captured in the protocol were inductively identified during photo coding. Through a constant comparative method, the team identified instances that either did not correspond to any existing indicator or revealed inconsistencies in existing definitions^(18)^. These cases were reviewed in team meetings and, when necessary, further discussed with representatives from Health Canada. As a result of this iterative process, the framework was updated – some indicator definitions were refined, and three new general indicators were developed: *appeals to taste*, *appeals to emotion* and cross*-referencing of marketing channels*.

For these general marketing indicators, we followed the protocol procedures in which food advertisement characteristics were coded as single indicators to avoid double-counting. For example, raters selected the best indicator to which to code the image of a smiling child (e.g. presence of children) and that attribute of the advertisement was not also coded to any other relevant indicator. Some marketing instances consisted only of plain sponsor or brand names, which met the definition of commercial promotion but did not satisfy the operational definitions of any Universal Indicator. Accordingly, these were counted as marketing instances but were assigned no marketing techniques. In addition, eleven sport-related indicators were inductively developed through pilot coding of 200 photos. The sport-related indicators were coded on top of the general indicators (e.g. an image of a child playing soccer could be coded as *presence of children* as well as *people or characters engaging in physical activity)*. Full definitions of all indicators are provided in online supplementary material, Supplemental Table S1.

### Classification of healthfulness

Each marketing instance was classified as promoting a product, brand or retailer, following procedures established in prior studies^(17)^. Classifications were based on Health Canada’s unpublished protocol *Monitoring Food Marketing to Children: A Protocol for Classifying Foods* (2021), which evaluates products as ‘of concern’ or ‘not of concern’ based on the presence of added sugars, saturated fats and sodium. When the product was available in the University of Toronto’s Food Labeling Information Program database, its classification was recorded directly based on the proportion of ‘of concern’ products that exceeded cut-offs for added saturated fat, sugar and sodium^(19)^. If the product was not found, nutrient and ingredient information were obtained from manufacturer or retailer websites, and healthfulness was individually assessed by nutrient content. Thresholds for excess added sugars, saturated fats and sodium are provided in an online supplementary material, Supplemental Table S2. In cases where nutrition information was insufficient, products were classified using generic category-based classifications outlined in the protocol. Brands and retailers were classified using the University of Toronto’s Menu-Food Labeling Information Program database, based on the proportion of listed products classified as ‘of concern’. Menu-Food Labeling Information Program database is Canada’s most comprehensive database of packaged food and beverage nutrition information. Those with more than 50 % of products falling into this category were themselves coded as ‘of concern’. If a brand or retailer was not included in the Menu-Food Labeling Information Program list, classification was not possible (*n* 346). Instances involving grocery, agricultural or alcohol brands were excluded from healthfulness classification (*n* 384). A final sample of 1846 marketing instances was analysed for healthfulness.

### Statistical analysis

Descriptive statistics were used to summarise the frequency and distribution of marketing techniques. Chi-square tests were employed to examine associations between categorical variables, including the co-occurrence of general and sport-related techniques, and the relationship between marketing indicators and product healthfulness. All statistical tests were conducted using SPSS, with significance set at *P* < 0·05.

## Results

### Prevalence of marketing techniques

Among the 2576 marketing instances analysed, 2354 (91·4 %) included at least one general marketing technique. Among instances with at least one general technique, the mean (sd) number used was 1·9 (1·2), with a range of one to nine techniques. Sport-related marketing techniques were less commonly observed. Only 309 (12·0 %) marketing instances featured at least one sport-related technique, whereas 1958 (89·3 %) showed none. Among those with any sport-related techniques, the mean (sd) number used was 1·8 (0·8), with a range of 1 to 7 techniques.

**Figure 1.**
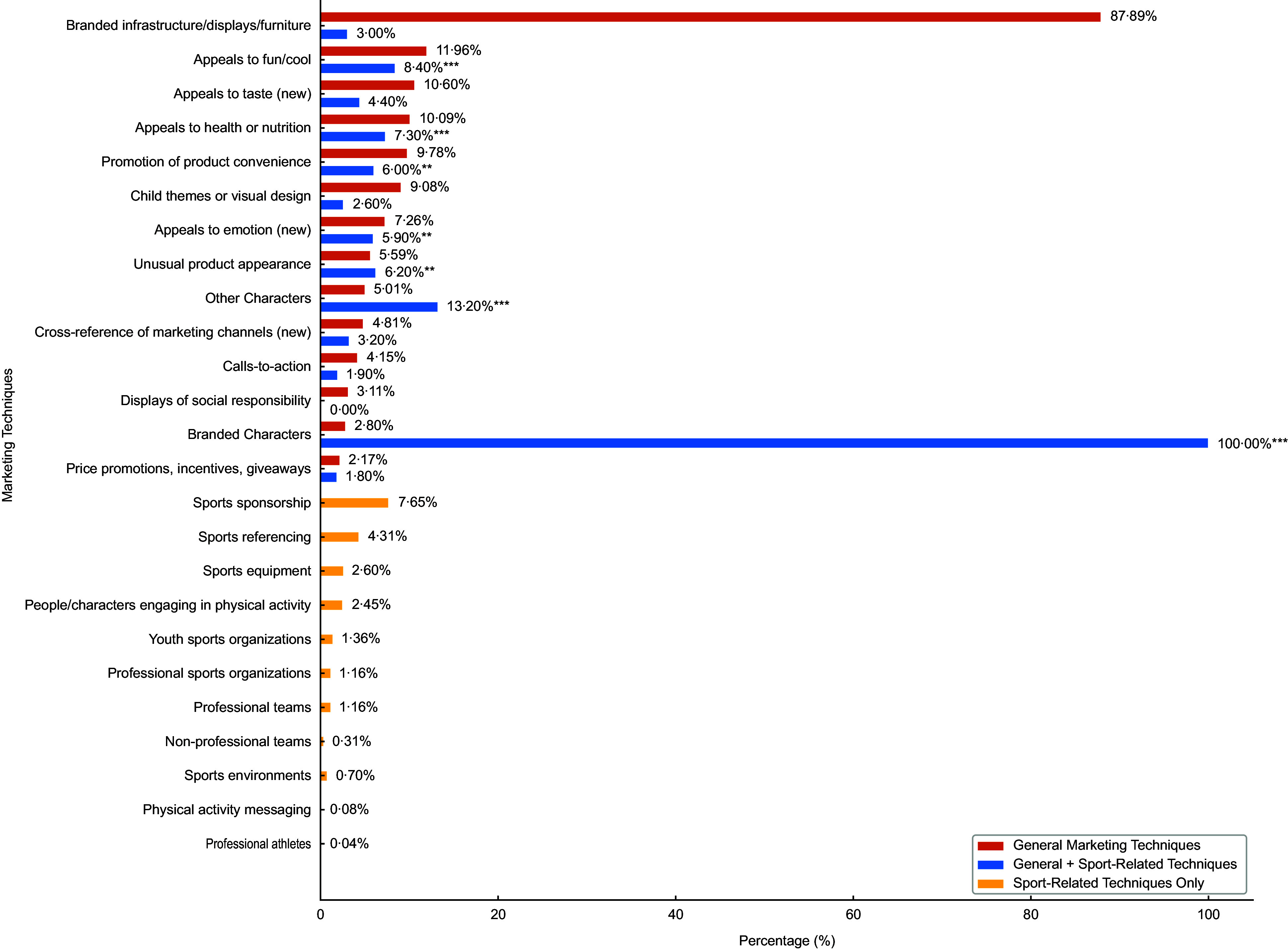
Prevalence of selected general and sport-related marketing techniques in food and beverage promotion across Canadian recreation and sport facilities (*n* 2576). The figure includes fourteen general techniques that appeared in at least 1 % of instances featuring any general content (*n* 2354) and all eleven sport-related techniques. Data labels indicate the proportion of total marketing instances in which each technique was observed. *Chi-square test results are significantly associated between the corresponding general technique and the presence of sport-related content (*P < 0·05, **P < 0·01, ***P < 0·001).

Figure 1 presents the frequency of marketing techniques (general and sport-related) that appeared in ≥ 1 % of instances. *Branded infrastructure/displays/furniture* was the most frequently observed general technique (87·89 %), followed by *appeals to fun/cool* (11·96 %), *appeals to taste* (10·60 %), *appeals to health or nutrition* (10·09 %) and *promotion of product convenience* (9·78 %). Among sport-related techniques, *sports sponsorship* (7·65 %) and *sports referencing* (4·31 %) were the most common, followed by *sports equipment* (2·60 %) and *people/characters engaging in physical activity* (2·45 %). Detailed numeric data for all indicators are reported in online supplementary material, Supplemental Tables S3 and S4.

Due to the overwhelming presence of branding in the RSF, we investigated its use with other indicators. *Branded infrastructure, displays and furniture* occurred with at least one other marketing technique half of the time (*n* 991, 51·1 %). The marketing techniques most commonly used with *branded infrastructure, displays and furniture* were *appeals to fun/cool* (26·4 %), *appeals to taste* (23·5 %), *appeals to health and nutrition* (20·8 %), *child themes or visual designs* (20·6 %), *promotion of product convenience* (15·3 %) and *unusual product appearance* (13·7 %). Only 11·0 % (*n* 109) of *branded infrastructure, displays and furniture* included any sport-related indicators. The sport-related marketing technique, ‘*Sponsor’ references*, was only found in sixty-one *branded infrastructure, displays and furniture* (6·2 % of the marketing instances with branding; 55·7 % of marketing instances with branding and a sport-related technique).

### Association between general and sport-related content

Of all marketing instances, 279 (10·8 %) used both general and sport-related techniques, 2075 (80·5 %) used only general techniques and 30 (1·2 %) used only sport-related techniques. A chi-square test confirmed a significant association between the two (χ² = 17·97, *P* < 0·0001), indicating that sport-related techniques rarely appear in isolation but are more often embedded with general marketing strategies.

All *branded character* instances (100 %) were associated with sport-related marketing, indicating complete overlap between the two indicators (χ² = 2576·00, *P* < 0·001). Other general techniques were significantly associated with sport-related content including *other characters* (χ² = 53·89, *P* < 0·001), *appeals to fun/cools* (χ² = 41·05, *P* < 0·001), *appeals to health/nutrition* (χ² = 21·67, *P* < 0·001), *unusual product appearance* (χ² = 10·50, *P* = 0·001), *promotion of product convenience* (χ² = 10·25, *P* = 0·001) and *appeals to emotion* (χ² = 7·07, *P* = 0·008). No significant associations with sport-related marketing were observed for *child themes or visual design*, *cross-channel references*, *calls-to-action*, *price promotions*, *displays of social responsibility* or *appeals to taste* (all *P* > 0·05). Detailed chi-square test results for all indicators are reported in online supplementary material, Supplemental Table S5.

### Healthfulness of promoted products

**Figure 2.**
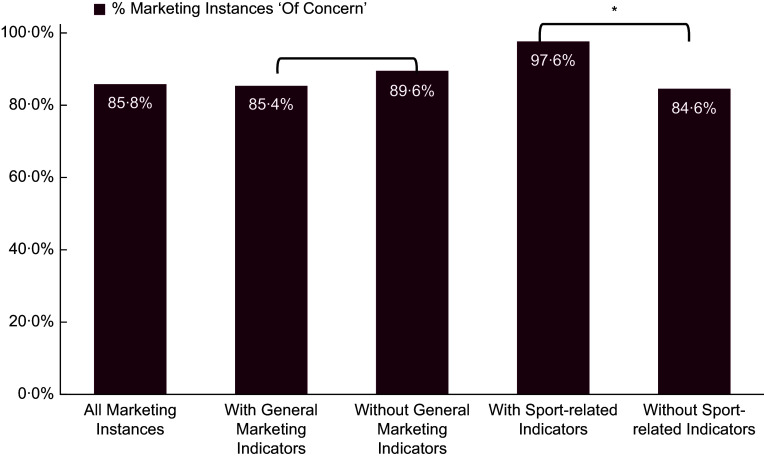
Healthfulness of marketing instances by general and sport-related techniques. Sport-related techniques were significantly associated with unhealthy products (*P < 0·001); no significant difference was found for general techniques.

The majority of food marketing instances in RSF were considered ‘of concern’ from a healthfulness perspective (85·8 %, *n* 1583) (Figure 2). General marketing techniques were present in 1702 instances; of these, 85·4 % were of concern, compared to 89·6 % of the 144 instances without general techniques (χ² = 1·88, *P* = 0·171) (Figure 2). This difference was not statistically significant. In contrast, sport-related marketing was significantly associated with unhealthier products: among 164 instances with sport-related content, 97·6 % were of concern, compared to 84·6 % among the 1682 without sport-related content (χ² = 20·54, *P* < 0·001) (Figure 2). These results suggest that less healthy products and brands more often include sport-related marketing. Full cross-tabulated results for each indicator are presented in online supplementary material, Supplemental Table S5.

## Discussion

This study examined the persuasive content of food and beverage marketing in a diverse sample of Canadian RSF. We aimed to quantify the use of general marketing techniques, assess the use of sport-related marketing techniques and evaluate the healthfulness of the promoted products/brands using these techniques. Food marketing instances recorded in RSF generally displayed at least one general marketing technique, with branding being the most frequently observed. It was often displayed on walls, floors, boards or displays (*branded infrastructure, displays and furniture)* and commonly paired with another technique half of the time. *Appeals to fun/cool*, *taste* and/or *health and nutrition* appeared in approximately 10–13 % of instances, while child-oriented visuals (*child themes or visual designs*) were present in nearly 10 %. Sport-related marketing was present on 12 % of marketing instances – most commonly through sponsor references – and was significantly more likely to co-occur with marketing techniques using characters (*branded characters*, *other characters*), as well as other techniques (*appeals to fun/cool*, *health/nutrition*, and *emotion*, *unusual product appearance*, and *price promotions, incentives and giveaways*). Further, ‘of concern’ food marketing instances were significantly more likely to include sport-related techniques than healthier products. Overall, there was no difference in healthfulness between marketing instances with and without general marketing techniques; however, most food marketing in RSF still promoted products considered to be ‘of concern’.

In our study, both general and sport-related marketing techniques commonly exhibited characteristics of indirect persuasion: marketing that influences consumer attitudes or behaviours through subtle emotional, sensory or symbolic cues, often without explicit messaging^(20)^. Many general techniques – such as brand visibility, appeals to fun or coolness and promotion of convenience – relied on emotional and sensory cues rather than conveying factual information about health or nutrition. Lacking clear behavioural prompts, these strategies are harder to detect through standard surveillance methods. Their subtle and embedded nature also makes them particularly difficult for children to recognise as marketing, limiting their ability to critically assess promotional content^(21)^. Over time, repeated exposures foster unconscious relationship-building between consumers and brands. This phenomenon, known as cultural camouflage, allows unhealthy products to blend into everyday life and gain social legitimacy by aligning with common cultural values, thereby reducing consumers’ awareness of persuasive tactics and increasing their acceptance of unhealthy food choices^(20,21)^.

Moreover, we observed differences in how these strategies were used across product types. General marketing techniques were used across both healthy and unhealthy products, with no clear pattern based on healthfulness. In contrast, sport-related strategies were predominantly used to promote less healthy items, reinforcing the well-documented sport health halo effect, where associations with physical activity mask the health risks of the promoted products. Notably, multiple techniques often appeared simultaneously within a single marketing instance, such as branding, emotional messaging, sport references and cross-platform prompts. This combination of strategic selectivity and persuasive layering is particularly concerning in environments frequented by children and warrants greater regulatory attention.

### General marketing techniques

Our results indicated that general marketing techniques were widespread in RSF and often appeared in combinations within a single promotional instance, rather than being used in isolation. Among the fourteen most frequently observed techniques, the majority were implicit in nature – focusing on brand visibility, emotional or sensory appeals, or promotion of convenience. These implicit strategies relied on cues such as happiness, belonging or taste, rather than factual claims about nutrition or health. Existing monitoring and regulatory frameworks typically focus on explicit cues – such as child-directed characters, health or nutrition claims, price promotions or product-based restrictions informed by nutrient profile models. This emotional framing can enhance persuasive power while remaining largely invisible to existing regulatory protocols^(11)^. The use of such bundled techniques reflects a deliberate strategy to construct a dense promotional environment, particularly in family-oriented recreational spaces. For example, branded infrastructure often appeared alongside appeals to fun, novelty or health messaging. Prior research on the cumulative effects of persuasive appeals supports this approach, suggesting that simultaneous exposure to multiple cues can intensify brand influence and reduce critical resistance among children and adolescents^(22,23)^. Rather than relying on any single message, these multi-layered strategies operate synergistically to shape perceptions – often making their influence more psychologically potent, even when individual elements appear benign^(23)^.

A key contribution of this study is the identification of three additional marketing techniques, not captured in the current monitoring protocol – *appeals to taste, cross-channel references* and *appeals to emotion*. These techniques reflect categories recognised in international monitoring frameworks. For example, the WHO framework on food marketing to children identifies emotional appeals (e.g. using fun, friendship or excitement) and sensory-based techniques (e.g. emphasising taste, enjoyment or craving) as persuasive strategies that increase the appeal of unhealthy food products and should be restricted^(12,24)^. In addition, the WHO framework highlights the need to address cross-platform digital engagement, which aligns with our indicator ‘cross-channel references’^(25)^. Similarly, the INFORMAS food promotion module includes indicators that capture emotional messaging, sensory-based content and multi-channel marketing strategies^(26)^.

The *appeals to taste* indicator referred to marketing that emphasises sensory characteristics such as flavour, freshness or texture (e.g. ‘delicious’, ‘freshly baked’ or ‘cool and refreshing’). In our study, *appeals to taste* were consistently observed in conjunction with other general techniques, particularly emotional appeals and branding (online supplementary material, Supplemental File 6 for example photo). This co-occurrence pattern differs from the other new indicators, which appeared independently in 69–82 % of instances. Prior research highlights taste as a primary driver of children’s food preferences and purchase intentions, suggesting that omitting this indicator may underestimate persuasive impact^(27)^.

*Appeals to emotion* refer to marketing strategies that evoke affective responses – such as pride, belonging, gratitude or inspiration – rather than rational or informational persuasion. In our study, these emotional appeals were expressed through patriotic slogans, community-oriented messaging and value-based branding, particularly on vending machines, posters or product packaging in RSF settings (online supplementary material, Supplemental File 6 for example photo). These strategies function as psychological anchors that reinforce brand loyalty and social legitimacy, particularly in the marketing of low-nutrition products. Prior research shows that emotional narratives – such as those centred on family, sportsmanship or social responsibility – are often used to create a ‘moral halo’ that softens perceived health risks and shifts attention toward affective alignment, thereby fostering consumer trust and brand identification^(28,29)^.

While emotional appeals are commonly used in advertising, we suggest that their specific expression in relation to Canadian sovereignty, local pride or community identity deserves greater attention given the current international political climate. These messages may carry added symbolic and social significance, particularly in RSF environments. Existing marketing classifications typically include emotional, rational, and moral appeals^(30)^, where emotional appeals often refer to humour or joy (captured in *appeals to fun/cool*), and moral appeals (captured in *displays of social responsibility*). However, the emotional strategies we observed go beyond humour-based or ethical framing. They include value-and identity-based messaging that fosters belonging, cultural alignment and perceived brand integrity. Therefore, we recommend that this indicator be considered for universal use across settings. Further investigation may be needed to understand what attributes would be considered emotion-appeals.

*Cross-channel references* refer to marketing techniques that guide consumers from one medium to another – for example, from physical advertisements to digital platforms such as websites, social media or mobile apps. In this study, such techniques were observed across various locations in RSF including vending machines, banners, posters and digital displays. Unlike the *call-to-action* indicator, which emphasises explicit behavioural prompts such as ‘order now’ or ‘scan to participate’, *cross-channel references* typically lack direct instructions. Instead, they use indirect elements – such as QR codes, platform logos or branded URLs – to subtly guide consumers toward digital platforms, extending brand engagement beyond the physical environment. Online supplementary material, Supplemental File 6 for example photos.

This technique reflects a key feature of marketing in the digital age: the convergence of online and offline environments. Offline exposures in RSF are increasingly designed to initiate online engagement, resulting in continuous cross-platform interaction. While current research and regulatory frameworks often treat digital and physical media as separate domains – focusing either on digital platforms like social media and streaming services^(31,32)^ or on community-based settings like grocery stores and outdoor signage^(10)^ – our findings indicate that modern marketing strategies often bridge these spheres.

Monitoring frameworks, such as WHO guidelines and INFORMAS protocols, have not yet systematically addressed this channel convergence. Much food marketing research is conducted in siloed marketing channels; cross-channel marketing and cumulative marketing exposures continue to be under-researched^(3)^. Separate indicators for digital and community settings may miss marketing efforts that begin offline but influence behaviour online. As cross-channel strategies become increasingly common, failing to account for such integrations may underestimate real-world exposure. Future surveillance efforts should better integrate cross-platform indicators, particularly in community environments where digital interaction is initiated through physical marketing touch points.

### Sport-related techniques

Our study found that sport-related marketing techniques were disproportionately used to promote unhealthy products and that general sport themes – rather than references to youth or professional athletes – were more commonly employed. This pattern suggests a strategic attempt to harness the symbolic power of sport to reframe low-nutrient products as compatible with health and activity. This pattern reflects a classic ‘sport health halo effect’, where associations with physical activity can obscure the nutritional risks of the advertised products^(33)^. In environments designed to promote well-being, such as RSF, the symbolic value of sport may falsely legitimise unhealthy food choices. This effect is particularly concerning for children and youth, who are especially receptive to symbolic and emotional cues in advertising^(34)^. Prior research has shown that sport-based imagery enhances engagement and brand affinity among young audiences, making it a potent but potentially misleading marketing tool^(35)^.

We found a higher use of general sports themes than specific references to youth or professional athletes, teams or leagues/organisations. If these indicators were used to assess more dynamic marketing instances (e.g. digital advertisements rather than static posters), the findings may be different. Notably, general marketing techniques were broadly applied across product types and showed no significant difference in their use between healthy and unhealthy products. In contrast, sport-based strategies appeared to serve a more targeted function, reinforcing symbolic associations with health and vitality that may be particularly persuasive in youth-oriented marketing environments. These findings point to the need for regulatory attention, especially as marketing shifts toward increasingly immersive digital platforms.

These findings suggest that national monitoring frameworks should more explicitly account for indirect and value-based marketing techniques that are not fully captured in current regulatory categories. To ensure policy relevance and practical implementation, guidance for RSF may need to emphasise flexible standards that allow facilities to move gradually toward healthier promotional environments. Updating federal surveillance tools (e.g. Health Canada’s monitoring protocols) and other research protocols to include refined indicators – and offering clearer direction on the use of sport-related imagery when promoting products ‘of concern’ – would strengthen alignment between regulatory goals and real-world marketing practices. Such refinements would better support both national and local decision-makers in assessing exposure and improving the nutrition environment in community recreation settings.

### Strengths and limitations

This study was a comprehensive analysis of marketing techniques in RSF using an established coding protocol designed for Canada. It included a diverse sample of randomly selected RSF from across Canada. All data were double coded for validation, and inductive coding and the creation of new unique indicators identified gaps in the coding protocol. This study has several limitations. First, while the coding captured a wide range of static visual content, it did not include dynamic interactions or user engagement with the advertising materials (e.g. scanning QR codes). Second, nutritional information was unavailable for approximately 28 % of the marketing instances (notably for restaurant brands), limiting the completeness of healthfulness analyses. Third, some types of embedded advertising – such as logos painted on ice rinks or athlete endorsements – were either hard to photograph or not consistently visible across facilities, which may have resulted in an underestimation of how frequently these techniques were used. Additionally, the cross-sectional design prevents causal inference regarding the influence of marketing techniques on consumer behaviour. Future studies could explore longitudinal tracking of marketing exposure in RSF, analyse engagement metrics across digital platforms and incorporate audience-centred perspectives to deepen understanding of how such marketing affects food choices over time.

### Conclusion

This project identified common marketing techniques used, along with patterns in sport-related marketing techniques, and highlights gaps in monitoring frameworks for RSF. Overall, this project confirmed a pervasive presence of branding in food marketing instances in recreation and sport facilities, combined with another marketing technique half of the time. The use of marketing techniques was highly variable with only five techniques present in more than 10 % of food marketing instances. Sport-related marketing techniques were used in approximately 12 % of food marketing instances, of which the majority were references to sponsors. All branded character instances (100 %) appeared exclusively in sport-related marketing, indicating complete overlap between the two techniques. Other characters were also significantly associated with sport-related content. In general, almost all food marketing instances were considered ‘of concern’, but this was magnified when marketing instances included sport-related marketing techniques. Future research should explore food marketing comprehensively, including new indicators on taste, emotion and cross-referencing. Monitoring protocols for food marketing can be improved by ensuring subtle techniques, and reinforced messages across marketing channels are included. Our findings suggest that such protocols may benefit from a broader lens – one that also captures the more subtle, symbolic and affective strategies increasingly used in real-world food promotion.

## Supporting information

10.1017/S1368980026102377.sm001 Lei et al. supplementary material 1 Lei et al. supplementary material

10.1017/S1368980026102377.sm002 Lei et al. supplementary material 2 Lei et al. supplementary material

10.1017/S1368980026102377.sm003 Lei et al. supplementary material 3 Lei et al. supplementary material

10.1017/S1368980026102377.sm004 Lei et al. supplementary material 4 Lei et al. supplementary material

10.1017/S1368980026102377.sm005 Lei et al. supplementary material 5 Lei et al. supplementary material

10.1017/S1368980026102377.sm006 Lei et al. supplementary material 6 Lei et al. supplementary material

## Supplementary material

For supplementary material accompanying this paper, visit https://doi.org/10.1017/S1368980026102377
