# Supplementary material for: General and sport-related marketing techniques in Canadian recreation and sport facilities: cross-sectional photo analysis of food and beverage advertisements
Source: Public Health Nutr. 2026 Mar 26;29(1):e90. doi: 10.1017/S1368980026102377 (PMC13112309; doi:10.1017/S1368980026102377)
Supplement: Lei et al. supplementary material 1 — Lei et al. supplementary material [file S1368980026102377sup001.pdf]

Supplementary 5. Example Figures

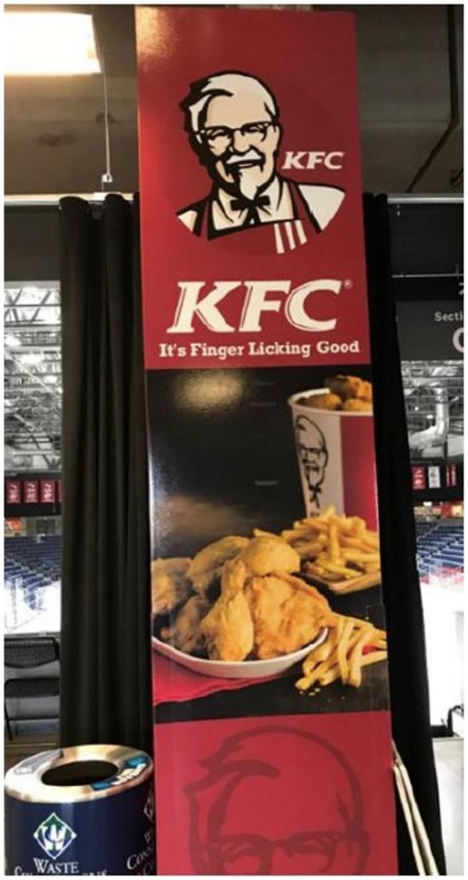

Figure 1. Examples of *appeals to taste* in RSF food marketing, featuring sensory-descriptive language (e.g."Finger licking good") and images (bunch of fried chicken and fries) with branding.

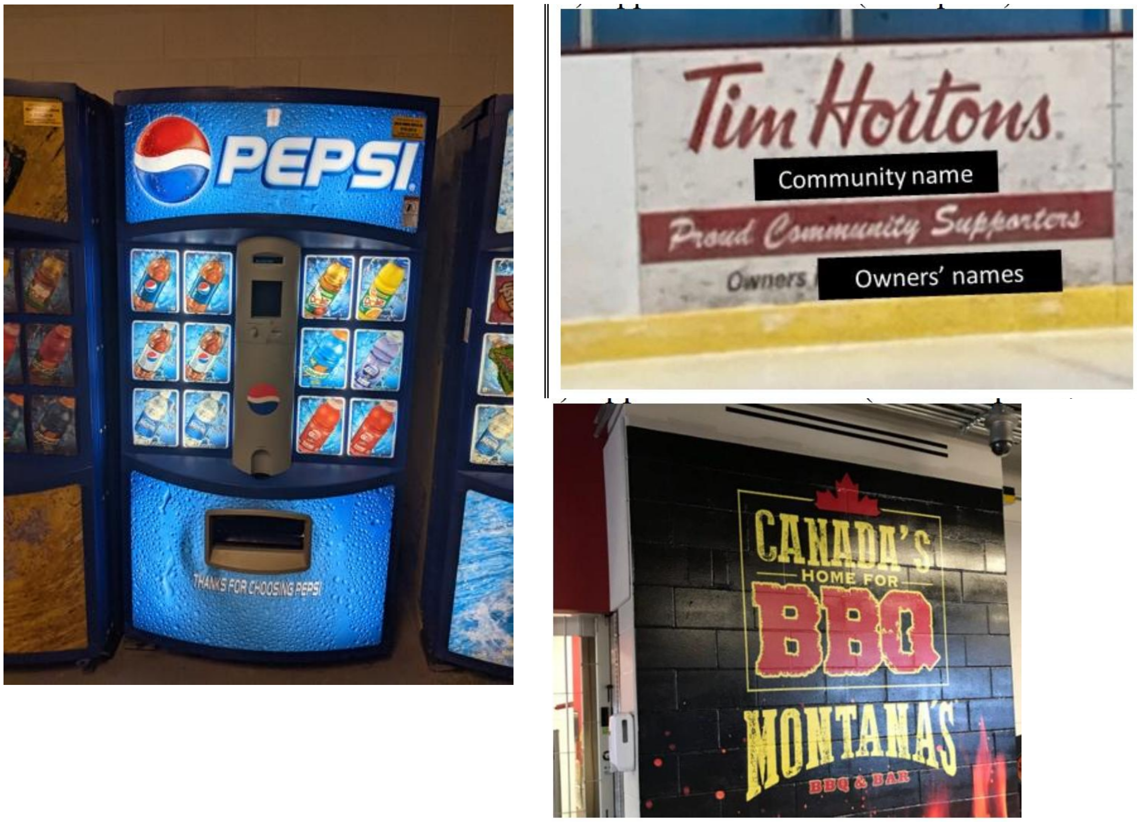

Figure 2. Examples of *appeals to emotion* in RSFs, including a) messages of appreciation (e.g., “Thanks for choosing Pepsi”); b) community-oriented messaging (e.g., “proud community supporters”); c) national symbols and messages (e.g. maple leaves, country’s name, "home of"). These messages do not reference specific products but associate the brand with positive emotions such as pride, connection, or gratitude—subtly influencing perception through affective alignment.

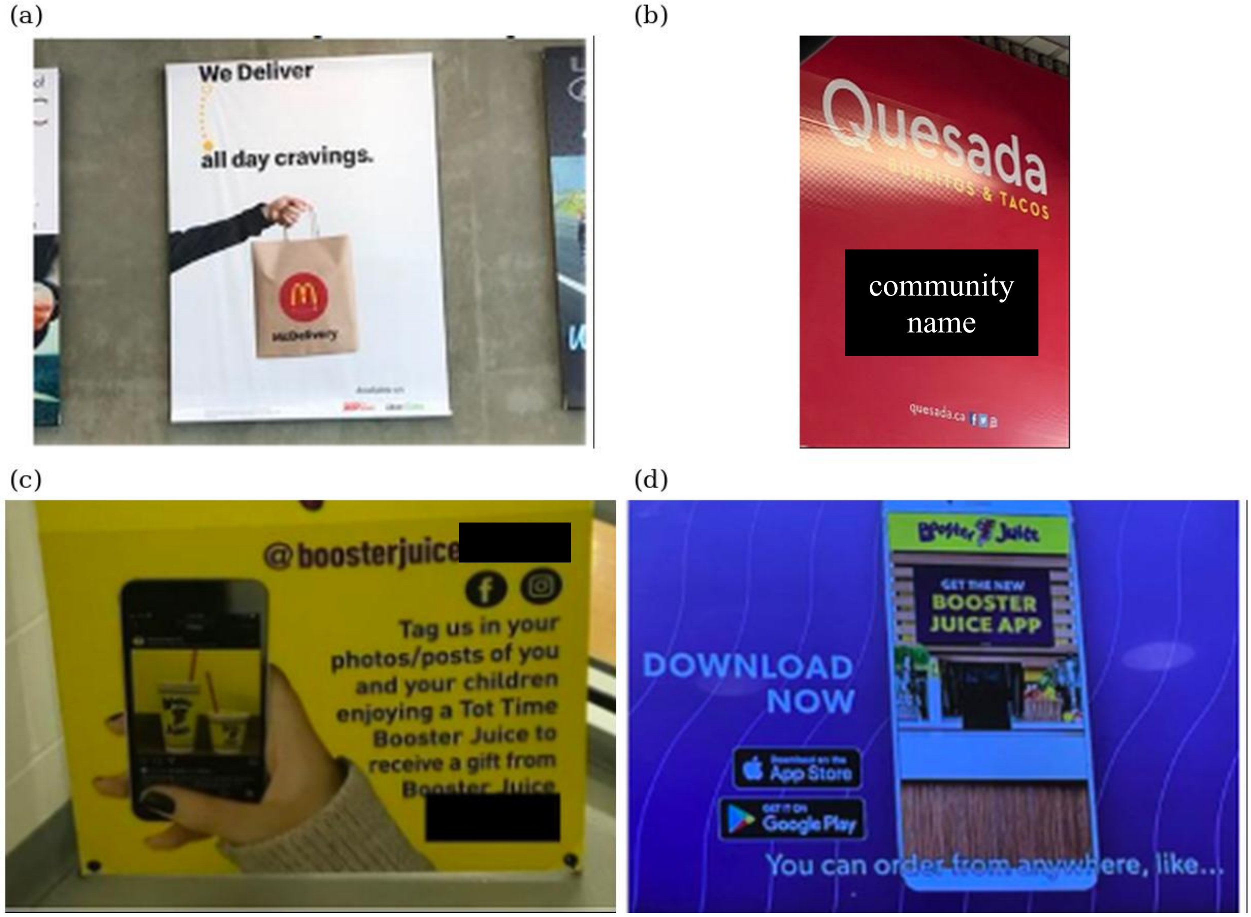

Figure 3. Examples of cross-channel marketing references observed in RSF environments. (a) McDonald’s poster displaying multiple third-party delivery platform logos in addition to its own McDelivery service; (b) Quesada wall sign simply featuring website URL and social media icons; (c) Booster Juice sign combining social media handles with a *call-to-action* message ("Tag us") inviting users to tag photos for rewards; (d) Booster Juice digital display encouraging app download with explicit *call-to-action* prompts ("Get the new Booster Juice app", "Download now") and platform icons (App Store and Google Play).
