# Supplementary material for: General and sport-related marketing techniques in Canadian recreation and sport facilities: cross-sectional photo analysis of food and beverage advertisements
Source: Public Health Nutr. 2026 Mar 26;29(1):e90. doi: 10.1017/S1368980026102377 (PMC13112309; doi:10.1017/S1368980026102377)
Supplement: Lei et al. supplementary material 2 — Lei et al. supplementary material [file S1368980026102377sup002.docx]

APPENDIX A – **Marketing Technique Descriptions and Revisions**

Table 1. Description of Universal Indicators, Adaptations and Explanations Based on Issues Experienced during Double Coding of Food and Beverage (F/B) Marketing Instances

|  | Indicator | Original Description | Updated description based on issues experienced during coding (see bolding) | Comments on Revision (Based on Coding Decisions) |
| --- | --- | --- | --- | --- |
| Universal Indicators  (All platforms and settings) | Presence of children | F/B ad features children (12 years and younger). | F/B ad features **real human** children (12 years and younger). | Only when real human children are depicted. Animated children, cartoon characters, or fictional creatures are not coded under this category and should instead be considered under “Other cartoon characters” if applicable. |
|  | Presence of teens | F/B ad features teens (13-17 years) | F/B ad features **real human** teens (13-17 years) | Same as Presence of Children |
|  | Adult-child situations | F/B ad features situations that play on the parent-child relationship or other authority-based relationship (e.g., coach-child, teacher-child). | F/B ad features situations that play on the parent-child relationship or other authority-based relationship **when highlighting adult-child interactions.** (e.g., coach-child, teacher-child, **family occasion, Mom’s Day dinner**). | Mentions of “family” and occasions like “Mom’s Day” will be coded under “Adult-child situations” when they highlight interactions between adults and children, such as family meals or a child presenting food to a parent. |
|  | Use of child language | F/B ad uses language that is associated with children, that is frequently used by children or that is directed at children | / | / |
|  | Use of teen language | F/B ad uses language that is associated with teens, that is frequently used by teens or that is directed at teens | / | / |
|  | Child themes/visual design | F/B ad uses themes, designs, colours, images, or other elements of audiovisual design that are commonly associated with children such as fantasy, magic, mystery, suspense, adventure, zoo animals, virtual worlds, etc. This could include references to, or the incorporation of, popular trends in children’s interests or preferences, which may vary year-to-year or based on geographic location.  *Note: this could refer to theme/design of the marketing instance (e.g., the side of a vending machine) or of the product itself (e.g., the packaging)* | F/B ad uses themes, designs, colours, images, or other elements of audiovisual design that are commonly associated with children such as fantasy, magic, mystery, suspense, adventure, zoo animals, virtual worlds, etc. This could include references to, or the incorporation of, popular trends in children’s interests or preferences, which may vary year-to-year or based on geographic location.  *Note: this could refer to theme/design of the marketing instance (e.g., the side of a vending machine) or of the product itself (e.g., the packaging).* ***While logos themselves are not coded, their presentation may be coded if it incorporates marketing techniques (e.g., special effects or unique styling).*** | Product logos will not be coded, but the presentation of the logo may include marketing techniques and thus its presentation can be coded. (e.g. if a logo is presented just as it would be on its website, no codes are applied. If the logo has a ‘special’ presentation or feature, that can be coded’). |
|  | Teen themes/visual design | F/B ad uses themes, designs, colours, images or other elements of audiovisual design that are commonly associated w ith teens such as themes linked to high school , socia lmedia , ‘hanging-out’ , popularit y, fashion, risk-taking, independence, etc. This could include references to, or the incorporation of, popular trends in teen’s interests or preferenc es, which may vary year-to-year or based on geographic location.  *Note: this could refer to theme/design of the marketing instance (e.g., the side of a vending machine) or of the product itself (e.g., the packaging)* | F/B ad uses themes, designs, colours, images or other elements of audiovisual design that are commonly associated w ith teens such as themes linked to high school , socia lmedia , ‘hanging-out’ , popularit y, fashion, risk-taking, independence, etc. This could include references to, or the incorporation of, popular trends in teen’s interests or preferenc es, which may vary year-to-year or based on geographic location.  *Note: this could refer to theme/design of the marketing instance (e.g., the side of a vending machine) or of the product itself (e.g., the packaging).* ***While logos themselves are not coded, their presentation may be coded if it incorporates marketing techniques (e.g., special effects or unique styling).*** |  |
|  | Unusual product appearance | The F/B ad depicts a product’s shape or colour that is unconventional or unusual for that type of product. | The F/B ad depicts a product’s shape or colour that is unconventional or unusual for that type of product (**e.g., candies shaped like recognizable objects [banana, stars]**). | Candies shaped like fruits, objects, or other non-traditional forms will be coded under "Unusual product appearance." |
|  | Unusual product flavour | The F/B ad promotes a product flavour that is unconventional or unusual for that type of product. | The F/B ad promotes a product flavour that is unconventional or unusual for that type of product (**e.g. ‘sour’ for candy**) | For candies, flavours will only be coded as “Unusual flavour” if they are considered unconventional. Common flavours such as fruit (e.g., strawberry, lemon, banana), vanilla, caramel, honey, mint, etc. will not be coded. However, descriptors such as “sour” that highlight a distinct taste experience will be coded as “Unusual flavour.” |
|  | Appeals to taste | **New indicator** | **Ad includes photos or text that emphasize the taste appeal of a food or beverage product. This may include images presenting the product in a ready-to-eat manner (e.g., juiciness, grill marks) or visual elements suggesting sensory attributes (e.g., water droplets for refreshment), as well as textual claims highlighting taste (e.g., "authentic Italian cuisine").** | Phrases that emphasize the sensory attributes of a product, such as "Powerful thirsty relief," will be coded under “Appeals to taste” if they highlight taste-related experiences, such as refreshment or cooling effects (e.g., “crisp,” “juicy,” “cooling”). |
|  | Appeals to  fun/cool | Ad makes appeals to the F/B being fun or funny, having fun while eating the product, humour or coolness/novelty5. This could include depictions of the food itself doing something fun, or depictions of the food in motion (e.g., a cookie diving into milk, or candies ‘exploding’ out of ice cream).  *Note: this could be as part of the product name (e.g. , “Fun Dip” , “Kool Kreatures”* )  *Note 2: there could be potential for overlap with other indicators, avoid double counting a single feature in the ad* | Ad makes appeals to the F/B being fun or funny, having fun while eating the product, humour or coolness, novelty, **or enjoyment**. This could include depictions of the food itself doing something fun, or depictions of the food in motion (e.g., a cookie diving into milk, or candies ‘exploding’ out of ice cream).  ***Note:*** ***Names of brands (e.g., Milk2Go, Save-on-Foods) and product lines (e.g., Powerade ION+) are not coded. However, slogans (e.g., "Finger lickin’ good") will be coded.*** | Names of brands (e.g. Milk2Go, Save-on-foods) and product lines (Powerade ION+) of brands are not coded, as they will not be regulated; Slogans (e.g. “Finger lickin’ good”) will be coded.  Phrases such as “Open Now” will be coded under “Promotion of product convenience” rather than “Novelty,” as they emphasize accessibility rather than uniqueness or newness. |
|  | Branded characters | F/B ad features company- or brand-owned characters or spokespersons. This could also include mascots of these characters.  *Note: these characters are often given a name and are used across marketing platforms* | / | / |
|  | Licensed characters | F/B ad features licensed characters from children’s and teens’ TV shows, movies, books, etc. | / | / |
|  | Other cartoon characters | F/B ad features generic cartoon characters, cartoon children/teens, animals, or imaginary/virtual creatures etc. that are not branded or licensed characters, celebrities, or cross-promotions to other media | **F/B ad features generic cartoon characters, including cartoon children/teens, animals, imaginary/virtual creatures, or anthropomorphized objects that are not branded or licensed characters, celebrities, or cross-promotions to other media.**  ***Note: Simple line drawings of objects without exaggerated or abstract features are not considered cartoon characters, but stylized human-like faces and objects with human-like features (e.g., a doughnut with legs) are included.*** | **The definition of cartoon characters need to better distinguish which elements should be coded. Simple line drawings of objects (e.g., a basic outline of a pig) are not considered cartoon characters, whereas line-drawn human faces and objects with human-like features (e.g., a doughnut with legs) are coded. Non-realistic human figures exaggerated or abstract animals, and anthropomorphized objects were explicitly included.** |
|  | Celebrities/public figures | F/B ad features actors, athletes, musicians, social media influencers, or other public figures | **/** | **/** |
|  | Cross-promotions | F/B ad features cross-promotions to movies/sports/TV show etc. other than one of the types of characters or celebrities described above. For example, the ad features aspects of a well-known fictional world, without specifically including the fictional characters.  *Note: these may appear in addition to the presence of any characters described above*  *Note 2: this includes sound effects in the ad that are clearly from movies or media* | **/** | **/** |
|  | Price promotions, incentives or giveaways | F/B is included as part of a price-promotion or premium, including discounted prices on other merchandise included with the purchase of a food or beverage product. Ad promotes contests, prizes, or giveaways available with or without purchase. | **F/B is included as part of a price promotion or premium, including explicit promotional wording (e.g., “Only $1.50,” “Special price”), discounts (e.g., “20% off”), bundle deals (e.g., “2 for 1”) or additional merchandise offered with the purchase of a food or beverage product (e.g. free drinks). Ad promotes contests, prizes, or giveaways available with or without purchase.**  ***Note:*** ***Simple price listings (e.g., “25 cents,” “Loonie,” “$1.50”) are not coded under "Price promotion."*** | Simple price listings (e.g., “25 cents,” “Loonie,” “$1.50”) will not be coded under "Price promotion." Only explicit promotional wording (e.g., “Only $1.50,” “Special price,” “2 for 1”) will be coded.  Names of brands (e.g. Save-on-foods) are not coded, as they will not be regulated; Slogans (e.g. “Big save on food”) will be coded. |
|  | Calls-to-action | F/B ad encourages consumers to buy the product, participate in a campaign, visit a product/brand/company website, social media or games-based brand website (including via QR code). Also includes the promotion of opportunities to “join”, “become a member”, complete a quiz, poll, or survey | F/B ad encourages consumers to buy the product, participate in a campaign, visit a product/brand/company website, social media or games-based brand website (including via QR code). Also includes the promotion of opportunities to “join”, “become a member”, complete a quiz, poll, or survey. **Ads must contain a clear action-oriented directive (e.g., “order,” “visit,” “follow,” “download”)** | The definition was refined to specify that a clear action-oriented verb must be present for an ad to be coded under Call-to-action. For example, a website URLs and social media icons alone are not sufficient to qualify as a Call-to-action; instead, these should be coded under Cross-referencing. Phrases such as "Download now" are coded under "Call-to-action." |
|  | Cross-referencing of marketing channels | **New indicator** | **Cross-reference of marketing channels includes any mention of a physical address for a food store, food retailer, or restaurant, as well as any reference to online platforms associated with food stores, retailers, or brands (e.g., social media pages, websites, mobile apps, company's name in the phone number). This also includes physical marketing materials (e.g., posters, billboards) that incorporate digital marketing features such as QR codes or links to online platforms.** |  |
|  | Appeals to health or nutrition | F/B marketing makes explicit appeals related to the healthfulness or nutritional quality of the product, its ability to promote wellness, growth, strength, or physical activity. This indicator also includes if the marketing makes implicit appeals to health or nutrition, such as the F/B being displayed alongside “healthy foods” (e.g., fresh fruit being depicted in an ad for breakfast cereal), or the F/B being shown consumed by children while participating in physical activity.  *Note: includes health and nutrition claims/symbols, as well as claims or symbols making reference to the product being organic or natural. This indicator is distinct from the packaging indicator below.* | F/B marketing makes explicit appeals related to the healthfulness or nutritional quality of the product, its ability to promote wellness, growth, strength, or physical activity. This indicator also includes if the marketing makes implicit appeals to health or nutrition, such as the F/B being displayed alongside “healthy foods” (e.g., fresh fruit being depicted in an ad for breakfast cereal), or the F/B being shown consumed by children while participating in physical activity. **In addition, this indicator includes any reference to healthy behaviors—such as eating habits, physical activity, sleep, or lifestyle choices that promote well-being (e.g., “eat well,” “balanced lifestyle”)—even if no specific food or brand is shown.**  *Note: includes health and nutrition claims/symbols, as well as claims or symbols making reference to the product being organic or natural. This indicator is distinct from the packaging indicator below.* | Any reference to healthy behaviors, including eating, physical activity, sleep, or other lifestyle factors that contribute to overall well-being (e.g., good eat, balanced diet) without any clear reference to a product or brand |
|  | Promotion of product convenience | Specific promotion of the product being convenient for snacking, packing in lunches, eating after sports practice, quickness, easy preparation, etc.  *Note: this does not include single-serve packaging (e.g., juice boxes or crackers and cheese packs) without specific promotion of their convenience* | Specific promotion of the product being convenient for snacking, packing in lunches, eating after sports practice, quickness, easy preparation, etc.  *Note: Single-serve packaging (e.g., juice boxes, crackers and cheese packs) and* ***brand names suggesting convenience (e.g., Milk2Go) are not coded*** *unless explicitly promoted for their convenience.*  ***Note2: Mentions of food delivery services count as "Promotion of product convenience." However, when referencing third-party delivery platforms (e.g., Skip The Dishes, DoorDash), they are coded under both "Cross-referencing" and "Promotion of product convenience," as they also highlight third-party platforms.*** | Phrases such as "Open Now" will be coded under "Promotion of product convenience" rather than "Novelty," as they emphasize accessibility rather than uniqueness.  Mentions of food delivery services (e.g., Skip The Dishes, DoorDash) will be coded under both "Cross-referencing" and "Promotion of product convenience." These references highlight third-party platforms while also emphasizing ease of access to the product. |
|  | Displays of corporate social responsibility | F/B marketing makes appeals to sustainability, philanthropy or contributing to other social causes | / | Sponsorship is not coded as “Corporate social responsibility” unless it explicitly promotes a brand’s commitment to social good beyond brand visibility (e.g., highlighting community support). |
|  | Appeals to emotion | **New indicator** | **F/B ad evokes emotional responses by appealing to sentiments such as patriotism, gratitude, nostalgia, or a sense of human connection (belongingness), thankfulness, or self-worth. This includes phrases that express appreciation (e.g., "Thank you for coming"), emphasize dedication or service (e.g., "Proud to serve you"), or invoke cultural, community, or patriotic identity.** |  |
| Specific Indicators for Restaurants | Children's menu | F/B is featured on a menu specifically designated for children | / | / |
|  | Children's activity | F/B is featured on a children’s activity sheet or other children’s materials provided at a restaurant | / | / |
|  | Value menu | F/B is featured on the “dollar” or value menu | / | / |
|  | Seasonal or limited time menus | F/B is featured as part of a seasonal, holiday-specific, or limited time promotion menu | / | / |
| Specific indicators for Community Settings | Specific indicator for Community Settings - Branded infrastructure, displays, furniture | Presence of infrastructure displays or furniture that are clearly associated with a F/B brand, either by way of shape, size, or appearance. This could include branded tents, flags, posters, kiosks, trucks, trash cans, scoreboards, bleachers, etc. | Presence of infrastructure displays or furniture that are clearly associated with a F/B brand **through its shape, size, distinctive design, or visible branding elements (e.g., logos, brand colors, or product imagery)**. This could include branded tents, flags, posters, kiosks, trucks, trash cans, scoreboards, bleachers, etc. | Branding on vending machines is categorized under "Branded infrastructure, displays, furniture," not "Branded clothing, objects, equipment."  Branded posters and standalone signs that have a clear commercial intent (e.g., menu boards with Pepsi logos, schedule boards with McDonald’s logos) are coded as "Branded infrastructure, displays, furniture." If the focus is on the brand itself, they are also coded under "Branding." However, posters primarily serving other purposes (e.g., a youth swimming event poster with a Save-on-Foods logo) are not considered branding. |
|  | Specific indicator for Community Settings - Branded clothing, objects, equipment." | Presence of clothing, objects that are clearly associated with a F/B brand, either by way of shape, size, or appearance. This could include sports jerseys, equipment, balls, medals, trophies, water bottles, etc. | / | / |
| Specific indicators for Digital Media | Viral marketing | F/B ad prompts viewers to engage with the brand by commenting, replying, sharing information with their peers (peer-to-peer marketing), re-posting content to their own feeds, tagging friends or using specific hashtags. | F/B ad prompts viewers to engage with the brand by commenting, replying, sharing information with their peers (peer-to-peer marketing), re-posting content to their own feeds, tagging friends or using specific hashtags.  ***Note:*** ***Hashtags and social media handles that encourage user interaction (e.g., "#ShareWithYourFriends") are coded under "Viral marketing." However, social media platform logos (e.g., Facebook, X, Instagram) without engagement prompts are coded under "Cross-marketing."*** | Hashtags and social media handles that encourage user interaction (e.g., "#ShareWithYourFriends," "@BrandName," "Like us on Facebook") will be coded under "Viral marketing." However, the presence of social media platform logos (e.g., Facebook, X, Instagram) without engagement prompts will be coded under "Cross-marketing." |
|  | Special effects | F/B ad features special effects, fast-cutting or animation | / | / |
|  | Songs or music | F/B ad features songs, music, jingles, or other audio that are child-like, such as with child voices, kids playing music, story-telling songs, lullabies, etc. | / | / |
