# Supplementary material for: General and sport-related marketing techniques in Canadian recreation and sport facilities: cross-sectional photo analysis of food and beverage advertisements
Source: Public Health Nutr. 2026 Mar 26;29(1):e90. doi: 10.1017/S1368980026102377 (PMC13112309; doi:10.1017/S1368980026102377)
Supplement: Lei et al. supplementary material 3 — Lei et al. supplementary material [file S1368980026102377sup003.docx]

Supplementary 2 – Nutrient Cut-offs for classification of Healthfulness

| **Established Thresholds for Added Saturated Fat, Sodium, and Sugars** | | |
| --- | --- | --- |
| **Nutrient** | **Thresholds for foods** | **Thresholds for main dishes with a reference amount (RA) above 200g** |
| **Saturated Fat** | A total of 2 g saturated fatty acid (SFA) per RA  or serving of stated size, whichever is the greater  **and**  ≤ 15% energy from the SFA | A total of 2 g SFA per 100g  **and**  ≤ 15% energy is from the SFA |
| **Sodium** | 140 mg per RA or serving of stated size whichever is the greater  **or**  140 mg per 50 g of the product if the RA is ≤ 30g or 30 mL | 140 mg per 100g |
| **Sugars** | 5 g per RA or serving of stated size whichever is the greater  **or**  5 g per 50 g of the product if the RA is ≤ 30g or 30 mL | 5 g per 100g |

Reference amounts (RA) can be found here: <https://www.canada.ca/en/health-canada/services/technical-documents-labelling-requirements/nutrition-labelling-table-reference-amounts-food.html>

Reference:

Health Canada. Monitoring food marketing to children: A protocol for classifying foods. Working Draft. December 2021. Unpublished.
