# Supplementary material for: General and sport-related marketing techniques in Canadian recreation and sport facilities: cross-sectional photo analysis of food and beverage advertisements
Source: Public Health Nutr. 2026 Mar 26;29(1):e90. doi: 10.1017/S1368980026102377 (PMC13112309; doi:10.1017/S1368980026102377)
Supplement: Lei et al. supplementary material 4 — Lei et al. supplementary material [file S1368980026102377sup004.docx]

Supplementary 3. Prevalence of General Techniques in Canadian Recreation and Sport Facilities

| Indicator | Count | Percentage of All Instances  (%, N=2576) | Percentage of Instances with Universal Techniques  (%, N=2354) |
| --- | --- | --- | --- |
| Branded infrastructure/displays/furniture | 2264 | 87.89% | 96.18% |
| Appeals to Fun/Cool/Novelty/Enjoyment | 308 | 11.96% | 13.08% |
| Appeals to Taste (New) | 273 | 10.60% | 11.60% |
| Appeals to Health or Nutrition | 260 | 10.09% | 11.05% |
| Promotion of Product Convenience | 252 | 9.78% | 10.71% |
| Child Themes or Visual Design | 234 | 9.08% | 9.94% |
| Appeals to Emotion (New) | 187 | 7.26% | 7.94% |
| Unusual product appearance | 144 | 5.59% | 6.12% |
| Other characters | 129 | 5.01% | 5.48% |
| Cross-Reference of Marketing Channels (New) | 124 | 4.81% | 5.27% |
| Calls-to-Action | 107 | 4.15% | 4.55% |
| Displays of social responsibility | 80 | 3.11% | 3.40% |
| Branded characters | 72 | 2.80% | 3.06% |
| Price promotions, incentives, giveaways | 56 | 2.17% | 2.38% |
| Use of Child Language | 21 | 0.82% | 0.89% |
| Adult-child situations | 19 | 0.74% | 0.81% |
| Games or Activities | 11 | 0.43% | 0.47% |
| Branded clothing, objects, equipment | 10 | 0.39% | 0.42% |
| Viral marketing (digital media) | 10 | 0.39% | 0.42% |
| Presence of Children | 6 | 0.23% | 0.25% |
| Seasonal or Limited Time Menus | 5 | 0.19% | 0.21% |
| Licensed characters | 4 | 0.16% | 0.17% |
| Unusual product flavour | 3 | 0.12% | 0.13% |
| Celebrities or Public Figures | 2 | 0.08% | 0.08% |
| Teen Themes or Visual Design | 2 | 0.08% | 0.08% |
| Children’s menu | 2 | 0.08% | 0.08% |
| Presence of Teens | 2 | 0.08% | 0.08% |
| Use of Teen Language | 1 | 0.04% | 0.04% |
| Adult-teen situations | 1 | 0.04% | 0.04% |
| Value menu | 1 | 0.04% | 0.04% |
| Children’s activity | 0 | 0.00% | 0.00% |
| Cross-promotions | 0 | 0.00% | 0.00% |
| Special effects | 0 | 0.00% | 0.00% |
| Songs or Music | 0 | 0.00% | 0.00% |
