# Supplementary material for: General and sport-related marketing techniques in Canadian recreation and sport facilities: cross-sectional photo analysis of food and beverage advertisements
Source: Public Health Nutr. 2026 Mar 26;29(1):e90. doi: 10.1017/S1368980026102377 (PMC13112309; doi:10.1017/S1368980026102377)
Supplement: Lei et al. supplementary material 5 — Lei et al. supplementary material [file S1368980026102377sup005.docx]

Supplementary 4. Frequency of Sport-Related Marketing Techniques in Food Marketing Instances

| Sport-Related Technique | Count (n) | Percentage of All Marketing Instances  (%, n=2576) | Percentage within Sport-Related Instances  (%, n=309) |
| --- | --- | --- | --- |
| Sports sponsorship | 197 | 7.65% | 63.75% |
| Sports referencing | 111 | 4.31% | 35.92% |
| Sports equipment | 67 | 2.6% | 21.68% |
| People/characters engaging in physical activity | 63 | 2.45% | 20.39% |
| Youth sports organizations | 35 | 1.36% | 11.33% |
| Professional sports organizations | 30 | 1.16% | 9.71% |
| Professional teams | 30 | 1.16% | 9.71% |
| Non-professional teams | 8 | 0.31% | 2.59% |
| Sports environments | 18 | 0.7% | 5.83% |
| Physical activity messaging | 2 | 0.08% | 0.65% |
| Professional athletes | 1 | 0.04% | 0.32% |
