# Supplementary material for: General and sport-related marketing techniques in Canadian recreation and sport facilities: cross-sectional photo analysis of food and beverage advertisements
Source: Public Health Nutr. 2026 Mar 26;29(1):e90. doi: 10.1017/S1368980026102377 (PMC13112309; doi:10.1017/S1368980026102377)
Supplement: Lei et al. supplementary material 6 — Lei et al. supplementary material [file S1368980026102377sup006.docx]

Supplementary 5. Associations Between General and Sport-Related Marketing Techniques in Food Marketing Instances

| General marketing techniques | Sport-related marketing techniques | | | Chi-square | P value |
| --- | --- | --- | --- | --- | --- |
|  | No | Yes | Total |  |  |
|  | (n=2504) | (n=72) | (n=2576) |  |  |
| Branded infrastructure, displays, furniture | 2196 (97.0%) | 68 (3.0%) | 2264 | 2.99 | 0.84 |
| Appeals to fun/cool | 282 (91.6%) | 26 (8.4%) | 308 | 41.05 | **<0.001** |
| Appeals to taste (new) | 261 (95.6%) | 12 (4.4%) | 273 | 2.88 | 0.09 |
| Appeals to health or nutrition | 241 (92.7%) | 19 (7.3%) | 260 | 21.68 | **<0.001** |
| Promotion of product convenience | 237 (94.0%) | 15 (6.0%) | 252 | 10.25 | **0.001** |
| Child themes or visual design | 228 (97.4%) | 6 (2.6%) | 234 | 0.05 | 0.822 |
| Appeal to emotion (new) | 176 (94.1%) | 11 (5.9%) | 187 | 7.07 | **0.008** |
| Unusual product appearance | 135(93.8%) | 9 (6.2%) | 144 | 10.50 | **0.001** |
| Other characters | 112 (86.8%) | 17 (13.2%) | 129 | 53.89 | **<.001** |
| Cross-reference of marketing channels (new) | 120 (96.8%) | 4 (3.2%) | 124 | 0.09 | 0.765 |
| Calls-to-action | 105 (98.1%) | 2 (1.9%) | 107 | 0.35 | 0.768 |
| Displays of social responsibility | 80 (100.0%) | 0 (0.0%) | 80 | 2.37 | 0.1 |
| Branded characters | 0 (0.0%) | 72 (100.0%) | 72 | 2576.00 | **<.001** |
| Price promotions, incentives, giveaways | 55 (98.2%) | 1 (1.8%) | 56 | 0.22 | 0.532 |
